# Reducing carbon, enhancing care: the future of sustainable medicine

**DOI:** 10.3389/fphar.2025.1637531

**Published:** 2025-07-03

**Authors:** Alessio Provenzani, Mia Como, Gennaro Martucci

**Affiliations:** ^1^ Clinical Pharmacy Service, Mediterranean Institute for Transplantation and Advanced Specialized Therapies (IRCCS ISMETT), Palermo, Italy; ^2^ Department of Pharmacy and Therapeutics, University of Pittsburgh School of Pharmacy, Pittsburgh, PA, United States; ^3^ Department of Anesthesia and Intensive Care, Mediterranean Institute for Transplantation and Advanced Specialized Therapies (IRCCS ISMETT), Palermo, Italy

**Keywords:** sustainable medicines, environmental, clinical pharmacology, carbon foot print, enhancing care

The United Nations has identified that climate change is one of the most urgent issues of our time (United Nations, 2020). They advocate for sustainable development initiatives that emphasize efforts and policies aimed to lessen climate change (United Nations, 2025). In addition, the Paris Agreement serves as a binding policy highlighting international commitment to addressing climate change to limit temperature increases above 1.5°C by the end of the century (Adeyeye et al., 2022; United Nations Framework Convention on Climate Change, 2025).

While healthcare may not be the primary contributor to climate change and greenhouse gas (GHG) emissions, it is not exempt from the impacts of climate change and still plays a significant role in the issue. Pichler et al. estimated that the healthcare system accounts for 5% of the national CO_2_ footprint (Pichler et al., 2019; Or and Seppänen, 2024). Rodríguez-Jiménez highlighted that the majority of the emissions in the healthcare industry are considered Scope 3, which refers to indirect emissions that are outside of the organization’s direct control like the supply chain (Rodríguez-Jiménez et al., 2023). These Scope 3 emissions make up approximately 50%–75% of the total emissions related to healthcare. A large portion of these uncontrolled emissions originate from medical equipment and pharmaceuticals.

To lessen healthcare’s environmental impact, some studies have explored strategies to focus on the manufacturing and distribution of products (Booth et al., 2023). The World Health Organization (WHO) highlights a framework to help health systems transition into more sustainable, low-carbon organizations (World Health Organization, 2023). They focus on optimizing the use of resources to reduce greenhouse gas emissions. Additionally, hospitals and healthcare systems are prioritizing sustainable pharmacy practices such as exploring more environmentally friendly formulations of medications (Booth et al., 2023). Pharmacy is uniquely positioned to drive this greener transition while still ensuring quality patient care.

**FIGURE 1:**
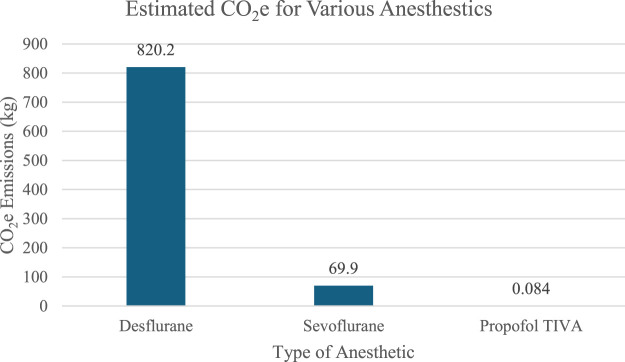
Calculated greenhouse gas emissions of a 7-h usage period of various anesthetics based on data from Allen and Baxter (2021).

Inhaled anesthetics and metered-dose inhalers (MDIs) have been identified to significantly contribute to the hospitals’ carbon footprint (Adeyeye et al., 2022; Or and Seppänen, 2024; Booth et al., 2023; Roy, 2021). Inhaled anesthesia gases are particularly harmful and contribute to 2% of National Health Service’s (NHS) carbon emissions (Adeyeye et al., 2022; NHS England and NHS Improvement, 2025). Nitrous oxide, specifically, is responsible for over half of anesthetic-related emissions and is the leading ozone-depleting agent (Adeyeye et al., 2022). Additionally, desflurane has been highlighted for its significantly higher greenhouse emissions compared to propofol. Allen and Baxter compared carbon dioxide equivalents (CO_2_e) for a 7-h usage period of inhaled anesthesia like desflurane and sevoflurane versus total intravenous anesthesia (TIVA) utilizing propofol (Allen and Baxter, 2021). As illustrated in Figure 1, their findings confirm that desflurane generates the highest CO_2_e (kg), followed by sevoflurane, while propofol TIVA has the lowest impact in terms of GHG. Adeyeye et al. also advocates for utilizing alternative anesthesia methods like regional anesthesia and TIVA. Adeyeye et al. (2022) examine various perspectives on how the pharmaceutical industry, healthcare providers, and patients can contribute to more sustainable medical practices. They highlight the need for collaboration between physicians and pharmacists in eco-pharmacy stewardship, where all healthcare professionals play a role in reducing environmental impact.

**FIGURE 2:**
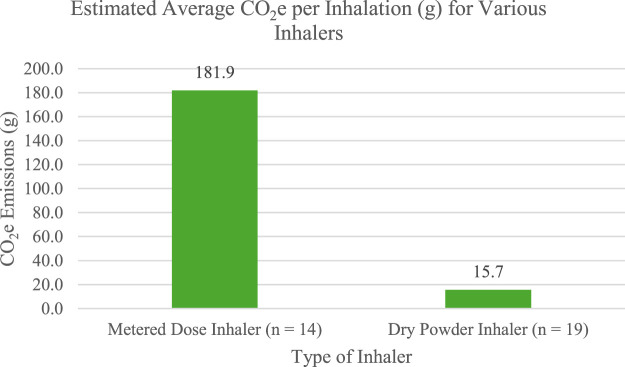
Average CO_2_e (g) per inhalation of various inhaler types based on data by Tirumalasetty et al. (2024).

Metered dose inhalers (MDI) also pose an environmental concern, contributing to approximately 3% of the NHS’s greenhouse gas emissions (Adeyeye et al., 2022; Or and Seppänen, 2024; NHS England and NHS Improvement, 2025). The main concern of metered-dose inhalers is the propellant, hydrofluoroalkanes (HFA), which are powerful greenhouse gases (Adeyeye et al., 2022). Strategies to reduce their environmental impact include increasing the use of propellant-free inhalers like dry-powder inhalers or improving patient education on the correct administration and disposal of MDI inhalers (Adeyeye et al., 2022; Or and Seppänen, 2024; NHS England and NHS Improvement, 2025). NHS cites similar recommendations for increasing the use of dry powder inhalers (DPI), improving disposal of previously used inhalers, and transitioning to the use of low-carbon propellant inhalers (NHS England and NHS Improvement, 2025). Despite, environmental improvement by lessening the use of MDI inhalers, there is still concern for patient usage when switching to an alternative inhaler. There are some cases where patients may require an MDI inhaler, including when they cannot produce a large enough inspiration to use the DPI appropriately (Adeyeye et al., 2022; Pernigotti et al., 2021). Despite the exception of specific patients who require the use of MDIs, DPIs are a more sustainable option. Tirumalasetty et al. analyzed the estimated greenhouse gas emission of various types of MDI and DPI inhalers (Tirumalasetty et al., 2024). Figure 2 displays the average CO_2_e (g) from 14 different MDIs and 19 different DPIs. Both categories of inhalers included a range of agents used to treat asthma and chronic obstructive pulmonary disease (COPD) (Tirumalasetty et al., 2024). Agents included inhaled corticosteroid (ICS) monotherapy, combination of long-acting muscarinic antagonist (LAMA) and long-acting beta agonist (LABA), ICS/LAMA/LABA, ICS/LABA, and short-acting beta agonist (SABA). The primary difference between groups was that the MDI category included a short-acting muscarinic antagonist (SAMA) monotherapy, Atrovent HFA (ipratropium), whereas the DPI category included monotherapy LABA agents such as the Serevent Diskus (salmeterol) and monotherapy LAMA agents like the Spiriva HandiHaler (tiotropium), Incruse Ellipta (umeclidinium), and Tudorza Pressair (aclidinium). Despite some variation in agents, both groups included a wide variety of inhalers with different medication types with the ability to compare CO_2_e between categories. Their analysis showed that the CO_2_e per inhalation (g) for all DPI inhalers was lower than that of any MDI inhaler. The highest emission reported from a DPI inhaler was 26.3 g whereas the lowest emission from an MDI inhaler was 49.5 g. With appropriate patient education and accessible alternatives to MDIs, physicians, pharmacists, and nurses can help to minimize inhalation’s impact on healthcare’s carbon footprint.

**FIGURE 3:**
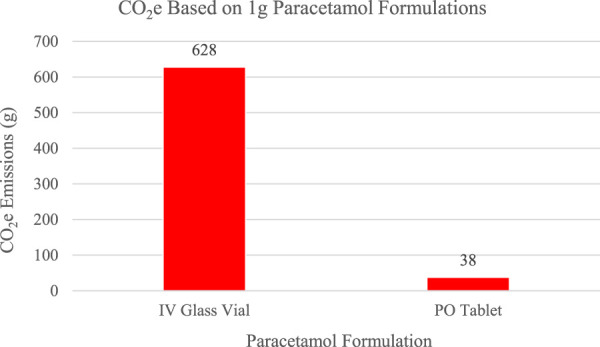
CO_2_e emissions from 1 g paracetamol based on a life cycle assessment by Davies et al. (2024).

Beyond MDIs and anesthesia, one additional approach involves a change from intravenous (IV) paracetamol to oral (PO) paracetamol for its potential to reduce pharmaceutical-related emissions (Davies et al., 2024; Myo et al., 2021; Hunfeld et al., 2024). As demonstrated by previous studies, transitioning from IV paracetamol in glass bottles to paracetamol tablets is an environmentally friendly solution to reduce healthcare greenhouse gas emissions overall. Paracetamol is commonly used in the ICU to manage pain. Suzuki et al. (2015) completed a life cycle assessment to compare the environmental impact of a PO tablet and an IV glass vial of paracetamol (Davies et al., 2024). They found that the total life-cycle greenhouse emissions from a 1 g dose of paracetamol was 38 g CO_2_e for an oral tablet and 628 g CO_2_e for an IV from a glass vial as shown in Figure 3. They further explain that the GHG emissions of the oral tablet were 16 times less than those from the glass vial. Myo et al. further emphasized that changing one-third of the IV paracetamol prescriptions to oral could result in five fewer tons of carbon emissions per year from production and disposal. Myo et al. (2021) described circumstances where one formulation of paracetamol may be more appropriate than the other, such as when a patient is not absorbing enteral feeds or if the patient is experiencing hemodynamic instability (Hunfeld et al., 2024). Aside from these patient-specific scenarios, PO paracetamol is a more environmentally friendly option with similar efficacy to the IV agent (Furyk et al., 2018).

Hospital pharmacy departments, with physicians and nursing teams, can implement several initiatives to support sustainable medication practices. Potential initiatives can highlight promoting low-carbon alternatives, implementing recycling programs for waste, and optimizing medication use overall. Hospital pharmacy teams can evaluate the use of glass IV agents and consider a switch to a more sustainable PO formulation, if clinically appropriate. Physicians and nursing teams can identify high-use medications in their respective units, assess areas of significant waste of medications, and create a more sustainable environment.

For our transplant patients and those with end-stage organ failure, a quality project was recently carried out at our Institute to assess the impact of using oral paracetamol compared to the injectable form—one of the most commonly used drugs in our post-operative patients.

Considering that a 1 g glass vial of paracetamol in 100 mL produces between 310 and 628 g of CO_2_, while tablets emit only 38 g (Davies et al., 2024), a team of nurses, doctors, and clinical pharmacists monitored paracetamol prescriptions over 2 weeks. It was observed that over 50% of parenterally administered paracetamol prescriptions could be switched to the oral formulation. When scaled over a year, these results could significantly reduce CO_2_ emissions. Additionally, the transition to an oral formulation could lower healthcare system costs, decreasing not only the cost of the drug, but also the cost of administration devices and the time required for managing intravenous infusions.

Collaboration between pharmacists, physicians, and nursing is essential to integrate and advance sustainability initiatives. Even small changes in drug formulations or product selection can help to significantly reduce healthcare’s impact on overall carbon emissions. By prioritizing sustainable alternatives, healthcare institutions can make a positive environmental impact. Pharmacists can lead the way in implementing these changes while still maintaining high-quality patient care.

Implementing sustainable practices in healthcare, particularly in pharmacy, is an opportunity to reduce the carbon footprint without compromising patient care. The reduction of inhaled anesthetic gases, adoption of alternatives to MDI inhalers, and transition from IV to PO paracetamol are just a few potential strategies to lessen healthcare’s environmental impact. Hospitals can take actionable steps toward a new environmentally friendly era by prioritizing low-carbon alternatives, optimizing medication utilization and disposal, and collaborating across healthcare teams. While healthcare may not be the largest contributor to climate change, it is still necessary to address the large contributions of emissions and promote more sustainable practices. Moving forward, healthcare institutions must commit to integrating these practices to ensure sustainability becomes a core component of everyday healthcare.
